# Designing a toolkit for the assessment of Health in All Policies at a national scale in Iran

**DOI:** 10.34172/hpp.2020.38

**Published:** 2020-07-12

**Authors:** Behzad Damari, Alireza Heidari, Maryam Rahbari Bonab, Abbas Vosoogh Moghadam

**Affiliations:** ^1^Department of Governance and Health, Institute of Neuroscience, Tehran University of Medical Sciences, Tehran, Iran; ^2^Health Management and Social Development Research Center, Golestan University of Medical Sciences, Gorgan, Iran; ^3^School of Public Health, Golestan University of Medical Sciences, Gorgan, Iran; ^4^Community-Based Participatory Policy Making, Islamic Parliament Research Center of the Islamic Republic of Iran, Tehran, Iran; ^5^Secretariat of Supreme Council for Health and Food Security, Ministry of Health and Medical Education, Tehran, Iran

**Keywords:** Health impact assessment, health policy, Health in All Policies (HiAP), Intersectoral collaboration, Social determinants of health

## Abstract

**Background:** Equitable promotion of health indicators requires cooperation among different sectors more than ever. The "Health in All Policies" (HiAP) approach contributes to this process through strengthening intersectoral collaboration. To implement this approach at a national scale, indicators of health-oriented performance from various organizations, and their measurement methods, need to be precisely defined. The aim of present study was to design a toolkit for implementing HiAP in Iran.

**Methods:** A review of literature and documents, as well as conducting semi-structured interviews and focus group discussions were undertaken to collect data for this qualitative study. Content analysis was applied to the collected data and the results were placed in three categories: criteria, sub-criteria and indicators; implementation processes; and implementation requirements.

**Results:** The toolkit aims to achieve various objectives, including intersectoral excellence and the systematic development of intersectoral collaboration. In the process section, reports on measures taken by organizations are assessed by a three-member audit committee. The top three organizations, in terms of intersectoral cooperation in achieving public health goals, are introduced in a Health Week. Requirements for success in achieving the HiAP approach include financial resources to implement the HiAP, a database, an electronic method for submitting reports, training courses, monitoring and annual reporting of relevant indicators, and formulating regulations in order to assess organizations.

**Conclusion:** Justification and training in various organizations to support the implementation of health-oriented measures, providing an annual ranking of organizations, and encouraging the organizations can contribute to the institutionalization of the toolkit through the SupremeCouncil for Health and Food Security. It is recommended that a Secretariat of sustainable development to be established under the Plan and Budget Organization (PBO) of the Islamic republic of Iran to monitor portfolio indicators.

## Introduction

Health is among the basic rights of every person in the community, and the government is required to provide it equitably and equally.^1^ In 1977, the ‘Health for All’ by the year 2000 strategy was adopted in the 30th session of the World Health Organization.^2^ “Intersectoral action for health” was emphasized in the Declaration of Alma-Ata and The Ottawa Charter supported “Healthy Public Policy”.^3^ According to the Charter, building healthy public policy is an action that “puts health on the agenda of policymakers in all sectors and at all levels, directing them to be aware of the health consequences of their decisions and to accept their responsibility for health”.^4,5^

The diversity of health determinants indicates that the Ministry of Health and Medical Education (MOHME) cannot provide and maintain public health on its own; achieving this objective at the national level requires intersectoral collaboration. As defined by the World Health Organization (WHO), intersectoral collaboration is “a recognized relationship between part or parts of the health sector with parts of another sector which has been formed to take action on an issue to achieve health outcomes (or intermediate health outcomes) in a way that is more effective, efficient or sustainable than could be achieved by the health sector acting alone.”^6^ Development of the concept of intersectoral collaboration in developed countries started several decades ago. In a report published by the Canadian Ministry of Health in 2007, the most successful pattern of intersectoral collaboration comprises both longitudinal and horizontal collaboration.^7^

The Health in All Policies (HiAP) approach, while concentrating on equity in health, states that all organizations and sectors have a role to play in public health.^8^ HiAP is a tool to support decisions which address the impact of all policies, seek synergy to simultaneously promote health and enhance public health and welfare, and benefit from structures, mechanisms and planned actions by sectors beyond the health sector.^7^ Most HiAP documents are prepared in developed countries with higher levels of welfare. This may be due to limitations in institutional and regulatory capacities in developing countries.^9^ This approach promotes health, improves lifestyle, increases productivity, enhances learning capacity, strengthens families and communities, enhances policymaker accountability, and supports sustainable development through emphasizing the impact of public policies on the health system.^10^ Global experience in implementation of the HiAP approach suggests that its strength lies in promoting health, equity and sustainability, while supporting intersectoral collaboration, benefiting multiple partners, engaging stakeholders, and creating structural or procedural changes. Therefore, HiAP is an approach that addresses the social determinants of health that are the main factors of health outcomes and inequity in health,^11^ as well as the effects of public sector decisions on health, considering the objectives of these determinants.^8^

According to Iran’s 4th five-year development plan, the Supreme Council for Health and Food safety was established as a structure to develop intersectoral collaboration.^12^ In this regard, a set of indicators was developed for the evaluation of intersectoral collaboration in health sector reform.^13^ Some of these indicators are Millennium Development Goals (MDGs) achievements, including the proportion of health-oriented objectives in the strategic plans of macro departments, improvements in indicators on healthy lifestyle and social behaviors, and the participation and membership rate of organizations’ experts in the main committees and councils of MOHME. Apart from the above-mentioned council, the Supreme Administrative Council was established according to Clause 10 of the Constitution of the Islamic Republic of Iran to achieve a decent administrative system in the first socio-economic development plan^14^ and regulations for the evaluation of executive organizations were implemented in 2003. However, evaluation of the health domain was not considered in the regulations. Furthermore, although the law related to evaluation of project-related environmental effects came into use in 1998,^15^ the concept of health impact assessments has become popular in recent years.^16^

Studies reflecting Iran’s status suggest that if the objective of health improvement were to be delegated to national organizations, materialization of the objective would follow a long road. Therefore, it is vital to evaluate the performance of national organizations. The present study aims at designing a toolkit for assessment of organizations’ health-oriented performance in line with implementing the HiAP approach at the national level.

## Materials and Methods

This qualitative study was conducted in three stages: review of literature and documents; semi-structured interviews; and focus group discussions.

### Review of literature and documents

Internet searches were performed on valid and accessible databases from 2000 to 2019. Domestic documents included previous interventions and experiences regarding successful assessments of organizational performance. The IRANDOC, Magiran, Islamic Parliament Research Center of Islamic Republic of Iran, SID, and Google Scholar databases were searched. The key words used to search domestic databases were organizations; assessment; executive organizations ranking; and assessment indicators of social accountability within organizations. A review of foreign sources emphasized evidence and experience gathered in implementing the HiAP approach. Searched databases were indexed in many studies and published journals such as PubMed, Scopus and Science Direct. In total, 160 scholarly articles and documents were found and reviewed. Given the purpose of this study, and after the screening and deletion of irrelevant articles and documents, 27 articles and nine documents were deemed suitable and were finally extracted.

### Semi-structured interviews

In conducting interviews, major institutions and units in Iran’s legislative, executive, and judicial systems were selected to evaluate the health-oriented performance of governmental organizations. Next, senior managers and experts working with the Islamic Consultative Assembly (law supervisor), Inspection Organization of Iran (assessment of progress and adherence to law by governmental organizations), Management and Planning Organization of Iran, Ministry of Interior (as the ministry with the greatest influence on managing social determinants of health), Ministry of Cooperatives, Labor, and Social Welfare and Iran Drug Control Headquarters (stewards of unemployment and addiction), Secretariat of Supreme Council for Health and Food Security, Provincial Secretariat of Health and Food Security, Academy of Medical Sciences (AMS), and National Institute of Health Research were interviewed. A purposive sampling approach was used. Participants’ experience and knowledge on the theme of the study, and their interest in participating, were applied as the inclusion criteria. Data were collected and analyzed simultaneously and sampling continued until data saturation was reached. Following 19 interviews, no new information was obtained, and previous data were repeated. Data were collected using interview guidelines comprising two main questions on the indicators of a respective organization’s health-orientation and assessment methods at the national level (including stewardship, process, requirements and use of outcomes). Appointments were made with selected individuals and interviews conducted in a suitable venue, so that interviewees could feel comfortable and relaxed.

### Focus group discussions

Three focus group discussions were held, with each attended by a public health expert, an organizational management expert, a representative and steward of organizational assessments, an economist and a sociologist. During these sessions, participants freely expressed their viewpoints and a session moderator guided the discussion. Collected indicators from the final stages were categorized.

Prior to the start of interviews and discussions, the purpose of this study was fully explained to participants, and interviews were recorded with the consent of interviewees, who were assured confidentiality. To achieve data accuracy, the valid Lincoln and Guba criteria were used. To ensure trustworthiness of data, credibility, transferability, dependability, and conformability were used.^17,18^ Participants’ reviews were used to verify data and codes. Initial codes, category extraction, and interview themes were provided to an external observer for investigation. Interview transcripts, codes, and extracted categories were provided to a research fellow and a number of faculty members who were familiar with the utilized manner of analyzing qualitative research but who did not participate in the study. They were asked to examine the validity of the data-coding process.

### Data analysis

Following transcription of the interviews, and through using an inductive approach, the content analysis method was applied for data analysis. This process included open coding, categorization and abstract construction. Finally, concepts from these two stages were merged and the complete content included criteria, sub-criteria and indicators, the implementation process and implementation requirements.

## Results

### Criteria, sub-criteria and indicators

In total, 14 main and eight contextual indicators were extracted. Criteria for HiAP assessment were as follows:

1. Intersectoral excellence, visible through health effects on consumers, employees and managers, along with environment health.
2. Systematic development of intersectoral collaboration in three forms: horizontal, vertical and time horizons.

Horizontal cooperation refers to collaboration among sectors with a similar objective at the decision-making level, while vertical cooperation is collaboration among a number of decision-making levels. Coordination in the time horizon is cooperation among agencies and organizations in the course of time, regardless of changes in executive political parties.

Table 1 shows the main criteria, sub-criteria and indicators for evaluation of HiAP.

### Implementation process

The three-member audit committee for the evaluation of organizations consists of the Secretary of the Supreme Health and Food Security Council (Ministry of Health and Medical Education) at the Secretariat of the Supreme Health and Food Safety Council of the country. The three main members include a representative of the Administrative Supreme Council of the country, a representative of the Planning and Budget Organization (PBO), and the head of the Secretariat of the Supreme Council for Health and Food Safety. Organizations’ reports on evaluation criteria are to be submitted to the Secretariat of the Supreme Health and Food Safety Council by the end of February of each year. A performance audit of each organization is carried out by Secretariat experts and a report prepared.

Organization performance evaluations are to be carried out at the end of the year through joint meetings with senior liaison officers of relevant organizations. The results of each meeting are summarized in accordance with the Secretariat’s audit report, and oral defense statements by senior liaison officers are submitted to the Supreme Council of Health and Food Security in the form of a final report. This is then submitted to the Administrative Supreme Council and the President. During Health Week, three top performing organizations are introduced.

### Implementation requirements

1. In the first year of evaluation, introducing and training senior liaison officers from relevant organizations is essential. The course content and/or syllabus must be in accordance with the curriculum as approved by the Administrative Supreme Council.
2. Upon the establishment of an organization’s sustainable development secretariat, that secretariat will monitor organizational activities in line with the country’s sustainable development goals.
3. The financial resources required for this evaluation are to be estimated by the Sustainable Development Secretariat in the first year of implementation and are to be included in the annual budget of the Ministry of Health and Medical Education and the Sustainable Development Secretariat. Funds come from tolls paid by producers who create products harmful to human health.
4. The Secretariat of the Supreme Council is required to provide a database and an electronic method for submitting reports.
5. The Secretariat of the Supreme Council of Health and Food Security is required to hold seminars, training courses, symposia and training modules for Secretariat members of relevant organizations. This task is to be facilitated by the National Institute for Health Research.
6. The National Institute for Health Research, in collaboration with the Statistical Center of Iran, is required to monitor relevant indicators in the health approach across all health policies and social components and to annually report all of these indicators to the central health audit committee for judgment. Indicator trends should be considered by the Statistical Center of Iran. These organizations must report indices annually to the audit committee so that the committee can judge potential trends.
7. The audit committee is required to formulate and disseminate regulations regarding evaluation of organizations in its first year.

## Discussion

Criteria for the evaluation of HiAP includes intersectoral excellence through examination of the impact of various factors on the health of consumers, employees and managers, as well as environmental health and the systematic development of intersectoral collaboration in three forms: horizontal, vertical, and time horizons. In the implementation process section, reports of the actions of related organizations are to be evaluated by a three-member audit committee and the top three performing organizations are to be introduced during Health Week. Implementation requirements include financial resources, a database and an electronic method for submitting reports, training courses, monitoring of relevant indicators and annual reporting, as well as formulation of the regulations required for an organization’s evaluation.

Baumann et al. and Baum et al. both presented evaluation frameworks for HiAP to encourage more responsive policymaking.^19,20^ Van Eyk et al indicated that HiAP in South Australia had dual goals; the facilitation of joined-up government for the common benefit of all citizens and consideration of the social determinants of health and inequities via cross-sectoral policies.^21^ The southern Australian experience in implementing HiAP shows that a focus on intersectoral collaboration and participation are requirements for implementation. In a report on the details of implementing an HiAP model to address regional migration in Adelaide, Australia in 2010, a tool entitled “Health Lens Analysis” was introduced, with the aim of maximizing health and wellbeing. This tool had five stages: urging organizations to commit to revising policies and agree on new policies; gathering evidence, literature reviews, data, and qualitative research; evaluation; preparing a final report containing a set of policy recommendations; and navigating and confirming reports and recommendations by the Health Department.^22^

In 2013, Gase et al presented seven intersecting actions for HiAP formulation and implementation: development of cross-sector collaboration; integrating health into organizational decision-making processes; strengthening human resources capabilities; coordinating budget and investment; evaluation and data systems; integrating research; synchronizing messaging and communications; and implementing accountability structures.^23^ Breton argued that the HiAP project faces two main impediments: low awareness within policy networks on the social determinants of health, as well as health actors’ neglect in investing in the complex problems of other sectors.^24^ Supportive factors for implementation of HiAP in Southern Australia include: the provision of a resource unit; establishment and maintenance of trust and credibility; and aligning HiAP with strategic priorities and core business.^25^

In 2003, Hillemeier et al published a set of indicators for every single social determinant of health. In other words, Hillemeier et al in their study, introduced various indicators, including: economic, political, educational, environmental, housing, transport, social psychology, social behavior and governance. If an organization successfully achieves these indicators, its performance is considered health-oriented.^26^ The results of the present study, in addition to stressing the intrinsic indicators of each organization, highlight the required collaboration in achieving macro and national indicators such as gross national product and life expectancy. This is a broader concept discussed in the syndemic model.^27^

A study conducted in Bristol in 2016 suggested that the Director of Public Health should work with Health and Wellbeing Board and other stakeholders to implement the prevention model to change modifiable unhealthy habits such as smoking, and look into HiAP.^7^

Molnar et al., showed that win-win strategies to implement HiAP include establishing a common language between different actors to facilitate communication; incorporating health in other policy programs; using dual results to appeal to the interests of different policy sectors; using scientific evidence to prove the effectiveness of the HiAP approach; and using health impact assessments to coordinate policies and attain public health outcomes.^28^

In comparison with other studies conducted on HiAP, the present study introduces a tangible tool in the form of a practical model for managers, health system experts and regulatory organizations to assess and compare the success of various organizations in implementing HiAP at the provincial and national levels. This model, i.e. the toolkit for evaluation of HIAP, may also be considered a self-assessment tool if an organization seeks to promote its performance.

A feature of this study is the possibility of integrating indicators into ongoing organizational processes without requiring new structures or imposing financial burdens, rather than stressing improved managerial methods.

In conducting the study, we faced some limitations. The proposed indicators in this study were input, process and output indicators, and achievement level indicators and their impact on those were not used. This can be a positive point, as impact indicators take a longer time to be visible and do not produce managerial motivation in the short term. Therefore, the criteria for an early return, including an organization’s success in training personnel, developing plans and allocating related budgets, may pave the way for cooperation and enhance sensitivity to HiAP in the early years.

## Conclusion

Utilizing the proposed toolkit for HiAP evaluation requires its integration into the current performance evaluation system of organizations, and would require the establishment of an audit committee in the High Administrative Council to evaluate the impact of organizations on health; however, this responsibility could be delegated to the Supreme Council for Health and Food Security and/or its secretariat. Organizing periodic courses and training staff in health-oriented actions, ranking organizations annually and providing encouragement from the Supreme Council for Health and Food Security are essential. Indicators could be revised after three years. It is also recommended a secretariat of sustainable development be established to monitor portfolio indicators, as well as economic, social and environmental indicators. To facilitate environmental, health and social impact assessment, it is suggested these be incorporated into a comprehensive assessment plan entitled: “National projects, plans, and politics of sustainable development” impact assessment.

In order to facilitate law enforcement, it is suggested environmental, health and social annexes be integrated into a comprehensive evaluation entitled: “sustainable policy development of national policies, programs and projects.”

## Ethical approval

The present study has been approved by Iran National Institute of Health Research. The participants were briefed about the purpose of the research and were assured on the confidentiality of information. Prior to data collection, verbal consent was obtained.

## Competing interests

The authors declare that there are no conflicts of interest.

## Funding

The present work has been financially supported by Iran National Institute of Health Research, Tehran, Iran.

## Authors’ contributions

BD: Conception and design, supervision, data collection, manuscript preparation, final proof of the manuscript. AH: Data analysis, Manuscript preparation, Final proof of the manuscript. NS: Data collection, Manuscript preparation, Final proof of the manuscript. AB: Data collection, Manuscript preparation, Final proof of the manuscript.

## Acknowledgments

The authors would like to express their appreciation and gratitude to Dr. Hadi Aiazi, Dr. Aliasghar Farshad, Dr. Narges Rostami Gooran and Shiva Mafimoradi in the office of the Deputy, MOHME for Social Affairs.

**Table 1:** Criteria, sub-criteria and indicators for evaluation of HiAP

**Themes** **(Main criteria)**	**Sub-themes** **(Sub-criteria)**	**Indicators**
Intrasectoral excellence	Consumers’ health, including six indicators	Developing policies for improvement of high-priority social determinants of health related to an organization’s field according to the 2025 vision, five-year plans, and sustainable development goals (SDGs)
		Organizational policies for equitable distribution of resources and opportunities according to an organization’s field
		Training the target group of an organization’s products or services about health orientation (related to the field of an organization)
		Having a system of participation by experts and people in the political cycle (design, execution, assessment)
		Execution of health-impact assessments
	Personnel and managers’ health	Having the financial resources and programs in place for health promotion in work settings
		Having all personnel covered by the ‘family physician’ program
	Environment health	Executing the environmental impact assessment law
		Having a charter and proclamation for environmental protection of managers and personnel related to an organization’s field
Intersectoral Collaboration	Horizontal	Signing mutual and/or multilateral memorandums of understanding (MOUs) and executing their content together with the Ministry of Health and Medical Education (MOHME)
		Effective criticism of health sector performance
		The effective role of an organization within the portfolio
	Vertical	Vertical cooperation for improvement of social determinants of health in the county, and at the provincial, national, regional and international levels
	Time horizon	Coordination in the time horizon (stabilization and institutionalization of continuous implementation of policies, with positive effects determined through evaluation, including the implementation of intersectoral agreements of previous administrations)
Contextual Indicators		Considering an organization’s vision plan; its share and role in provision, maintenance and promotion of public health should be considered a value and part of an overall social objective
		Drafting missives on health-orientation indicators
		Allocating special credits to health-oriented programs conducted in cooperation with the Ministry of Health and Medical Education (MOHME)
		Presenting annual reports to the Supreme Council for Health and Food Security referring to results, actions and indicator impact
		Ranking an organization’s subunits in terms of health orientation and the public announcement of results
		Participation of personnel in training courses on the principles and techniques of health orientation
		Quality and quantity of senior and specialized liaison officers within an organization who coordinate with the secretariat of the Supreme Council for Health and Food Security
		Having an active secretariat of sustainable development within an organization

## References

[1] Babazadeh Gashti A, Jafari N, Kabir MJ, Heidari A, Behnampour N, Honarvar MR (2016). Assessing rural family physicians performance according to healthcare managers, family physicians, and patients in Golestan province, Iran. Journal of Mazandaran University of Medical Sciences.

[2] Kabir MJ, Vatankhah S, Delgoshaei B, Ravaghei H, Jafari N, Heidari A (2013). Determinant criteria for designing health benefit package in selected countries. Life Sci J.

[3] Baum F, Delany-Crowe T, MacDougall C, Lawless A, van Eyk H, Williams C (2017). Ideas, actors and institutions: lessons from South Australian Health in All Policies on what encourages other sectors’ involvement. BMC Public Health.

[4] World Health Organization, International Conference on Health Promotion. Ottawa charter for health promotion: First international conference on health promotion, Ottawa, 21 November 1986. Geneva: World Health Organization; 1986.

[5] Ollila E (2011). Health in All Policies: from rhetoric to action. Scand J Public Health.

[6] Kreisel W, von Schirnding Y (1998). Intersectoral action for health: a cornerstone for health for all in the 21st century. World Health Stat Q.

[7] Stahl T, Wismar M, Ollila E, Lahtinen E, Leppo K. Health in All Policies: Prospects and potentials. Helsinki: Finland: Ministry of Social Affairs and Health; 2006.

[8] Damari B, Ehsani Chimeh E (2017). Public health activist skills pyramid: a model for implementing health in all policies. Soc Work Public Health.

[9] Kickbusch I (2013). Health in all policies. BMJ.

[10] Health in All Policies (HiAP) framework for country action. Health Promot Int 2014;29 Suppl 1:i19-28. doi: 10.1093/heapro/dau035. 25217354

[11] Rudolph L, Caplan J, Ben-Moshe K, Dillon L. Health in All Policies: A Guide for State and Local Governments. Washington, DC: American Public Health Association; 2013.

[12] Damari B, Vosoogh Moghaddam A, Bonak dari S. Improving approaches of intersectoral collaboration for health by health and food security high council in IR Iran. Journal of School of Public Health and Institute of Public Health Research 2014;11(3):1-16. [Persian].

[13] Beheshtian M, Olyaee Manesh A, Bonakdar S, Malek Afzali H, Larijani B, Hosseini L (2013). Intersectoral collaboration to develop health equity indicators in Iran. Iran J Public Health.

[14] Zare’ee MH. From human rights and respect to the “project to foster reverence in the high administrative councill”. Journal of Management and Development Process 2002;16(56-57):22-8. [Persian].

[15] Setudeh A, Ghoddosi F. The Necessity of Reviewing the Environmental Impact Assessment Process of Development Projects. National Conference on Environmental Impact; 2006; Tehran, Iran. [Persian].

[16] Fifth development plan of Islamic republic of Iran, 2010. Available from: http://isacmsrt.ir/files/site1/pages/barnamepanjom.pdf. Accessed 2019. [Persian].

[17] Shenton AK (2004). Strategies for ensuring trustworthiness in qualitative research projects. Education for Information.

[18] Eri M, Jafari N, Kabir MJ, Mahmoodishan G, Moghassemi MJ, Tahanian M, et al. Concept and challenges of delivering preventive and care services in prehospital emergency medical service: a qualitative study. Journal of Mazandaran University of Medical Sciences 2015;25(126):42-57. [Persian].

[19] Baum F, Lawless A, Delany T, Macdougall C, Williams C, Broderick D (2014). Evaluation of Health in All Policies: concept, theory and application. Health Promot Int.

[20] Bauman AE, King L, Nutbeam D (2014). Rethinking the evaluation and measurement of Health in All Policies. Health Promot Int.

[21] van Eyk H, Harris E, Baum F, Delany-Crowe T, Lawless A, MacDougall C. Health in All Policies in south Australia-did it promote and enact an equity perspective? Int J Environ Res Public Health 2017;14(11). 10.3390/ijerph14111288 PMC5707927 29068400

[22] Kickbusch I. Health in All Policies: The Evolution of the Concept of Horizontal Governance. In: Kickbusch I, Buckett K, eds. Implementing Health in All Policies: Adelaide 2010. Adelaide: Government of South Australia; 2010.

[23] Gase LN, Pennotti R, Smith KD (2013). “Health in All Policies”: taking stock of emerging practices to incorporate health in decision making in the United States. J Public Health Manag Pract.

[24] Breton E (2016). A sophisticated architecture is indeed necessary for the implementation of health in all policies but not enough: comment on “understanding the role of public administration in implementing action on the social determinants of health and health inequities”. Int J Health Policy Manag.

[25] Delany T, Lawless A, Baum F, Popay J, Jones L, McDermott D (2016). Health in All Policies in south Australia: what has supported early implementation?. Health Promot Int.

[26] Hillemeier MM, Lynch J, Harper S, Casper M (2003). Measuring contextual characteristics for community health. Health Serv Res.

[27] Tsai AC, Mendenhall E, Trostle JA, Kawachi I (2017). Co-occurring epidemics, syndemics, and population health. Lancet.

[28] Molnar A, Renahy E, O’Campo P, Muntaner C, Freiler A, Shankardass K (2016). Using win-win strategies to implement health in all policies: a cross-case analysis. PLoS One.

